# Myosin Regulatory Light Chain (RLC) Phosphorylation Change as a Modulator of Cardiac Muscle Contraction in Disease*

**DOI:** 10.1074/jbc.M113.455444

**Published:** 2013-03-25

**Authors:** Christopher Toepfer, Valentina Caorsi, Thomas Kampourakis, Markus B. Sikkel, Timothy G. West, Man-Ching Leung, Sara A. Al-Saud, Kenneth T. MacLeod, Alexander R. Lyon, Steven B. Marston, James R. Sellers, Michael A. Ferenczi

**Affiliations:** From the ‡ Molecular Medicine Section, National Heart and Lung Institute, Imperial College London, London SW7 2AZ, United Kingdom,; the ¶ Randall Division of Cell and Molecular Biophysics, Guy's Campus, King's College London, London SE1 1UL, United Kingdom,; the ‖ National Heart and Lung Institute, 4th Floor, Imperial Center for Translational and Experimental Medicine, Hammersmith Campus, Du Cane Road, London W12 0NN, United Kingdom,; the ** Structure and Motion Laboratory, Royal Veterinary College London, North Mymms AL9 7TA, United Kingdom,; the ‡‡ Cardiovascular Biomedical Research Unit, Royal Brompton Hospital, London SW3 6MP, United Kingdom,; the § Laboratory of Molecular Physiology, NHLBI, National Institutes of Health, Bethesda, Maryland 20892, and; the §§ Lee Kong Chian School of Medicine, Nanyang Technological University, 637553 Singapore

**Keywords:** Cardiac Muscle, Muscle, Myosin, Phosphorylation, Physiology

## Abstract

Understanding how cardiac myosin regulatory light chain (RLC) phosphorylation alters cardiac muscle mechanics is important because it is often altered in cardiac disease. The effect this protein phosphorylation has on muscle mechanics during a physiological range of shortening velocities, during which the heart generates power and performs work, has not been addressed. We have expressed and phosphorylated recombinant *Rattus norvegicus* left ventricular RLC. *In vitro* we have phosphorylated these recombinant species with cardiac myosin light chain kinase and zipper-interacting protein kinase. We compare rat permeabilized cardiac trabeculae, which have undergone exchange with differently phosphorylated RLC species. We were able to enrich trabecular RLC phosphorylation by 40% compared with controls and, in a separate series, lower RLC phosphorylation to 60% of control values. Compared with the trabeculae with a low level of RLC phosphorylation, RLC phosphorylation enrichment increased isometric force by more than 3-fold and peak power output by more than 7-fold and approximately doubled both maximum shortening speed and the shortening velocity that generated peak power. We augmented these measurements by observing increased RLC phosphorylation of human and rat HF samples from endocardial left ventricular homogenate. These results demonstrate the importance of increased RLC phosphorylation in the up-regulation of myocardial performance and suggest that reduced RLC phosphorylation is a key aspect of impaired contractile function in the diseased myocardium.

## Teaser

**Background:** Cardiac myosin regulatory light chain (RLC) phosphorylation alters cardiac muscle function.

**Results:** Phosphorylation affects mechanical parameters of cardiac muscle contraction during shortening.

**Conclusion:** Phosphorylation impacts mechanical function of cardiac muscle and is altered during cardiac disease.

**Significance:** Understanding RLC regulation by phosphorylation in cardiac muscle contraction is crucial for understanding changes in disease.

## Introduction

Interactions between the two contractile proteins actin (thin filament) and myosin (thick filament) drive the contractile machinery of muscle contraction (1). The molecular mechanisms by which these proteins are regulated in cardiac muscle contraction are still yet to be understood fully (2). Thin filament activation by Ca^2+^ binding to the troponin-tropomyosin complex is known to be important in normal cardiac contraction and in the dysfunction of contractile characteristics in heart failure (HF)^2^ (3). In contrast, thick filament proteins have less defined roles in regulation of cardiac contraction (4, 5).

Evidence suggests that myosin-associated regulatory proteins, such as the cardiac ventricular isoform of the regulatory light chain (RLC), have an influential role to play in muscle contraction mediated by phosphorylation (6–13). *In vitro* studies performed by Stull *et al.* (4) have shown a correlation between RLC phosphorylation and isometric force of twitch potentiation in skeletal muscle. This suggested that Ca^2+^ binding to troponin C (TnC) is not the only process that regulates striated muscle contraction. Furthermore, *in vitro* and structural studies have implicated the negative charge associated with phosphorylation of the RLC to structurally repel myosin heads away from the thick filament toward actin (14–16). There is also evidence that RLC phosphorylation may affect stiffness of the myosin lever arm (17) and/or hinge region in smooth muscle (18).

Furthermore, pathological mutations to the RLC in humans are known to present as familial hypertrophic cardiomyopathies. Many of these mutations occur in and around the phosphorylatable region of the RLC and can affect the ability of the RLC to be phosphorylated, as seen in the E22K mutation among others (12, 19, 20). Evidence also exists to suggest RLC hyperphosphorylation could drive hypertrophy (21).

Studies have been performed to elucidate RLC phosphorylation mechanisms; genetic mutant murine models of disease have been used, either replicating mutations found in human patients or creating mutant RLCs that are unphosphorylatable to assess calcium sensitivity changes (19, 22–26). Others have dephosphorylated RLC in cardiac preparations using 2,3-butanedione monoxime, which has unknown protein dephosphorylation specificity (14). These studies elucidated the effect a mutation has on cardiac pathology from model organisms but did not isolate the effect of RLC phosphorylation on muscle mechanics independent of other protein modifications. These studies did not assess mechanics during muscle shortening.

In this paper, a Phos-tag^TM^ SDS-PAGE method was utilized to observe the changing RLC phosphorylation profile during heart failure progression in human patients in New York Heart Association (NYHA)-classified HF progression and in a rat model of chronic MI, which manifests as early cardiac hypertrophy and eventual heart failure.

In addition, we studied and evaluated the mechanical effect of RLC phosphorylation on permeabilized cardiac tissue. We used force-velocity (FV) and power-velocity (PV) relationships to assess the effect a physiological range of RLC phosphorylations had on the contractile characteristics of permeabilized cardiac trabeculae. This was performed during muscle shortening over a set of velocities in which the heart generates power and performs work in the physiological range.

## EXPERIMENTAL PROCEDURES

### 

#### 

##### Rat MI Model

All animal surgical procedures and perioperative management were carried out in accordance with the Guide for the Care and Use of Laboratory Animals published by the United States National Institutes of Health under assurance number A5634-01. Adult male Sprague-Dawley rats (250–300 g) underwent proximal left anterior descending coronary ligation to induce chronic myocardial infarction as described previously (27). Following 4 or 16 weeks, rats were sacrificed by cervical dislocation. Age-matched controls were used as a comparison with two MI time points, 4 weeks post-MI and 16 weeks post-MI. Relative hypertrophy was assessed by heart weight to body weight ratio, and ejection fraction was measured by M-mode echocardiography (Vevo 770, Visualsonics) to give a measure of cardiac function (Table 1).

##### Myofibril Collection

10 mg of rat myocardium was removed from the left ventricular endocardial region close to the apex, frozen in liquid nitrogen, and stored at −80 °C. On the day of the experiment, samples were mechanically homogenized using a pestle and mortar cooled with dry ice. Samples were then added to SDS-PAGE sample buffer. Human tissue samples were also removed from the endocardial layer of the left ventricle and prepared the same way. Porcine hearts were obtained from the Royal Veterinary College. These were rinsed in ice-cold saline and prepared for gel analysis in the same manner as rat and human myofibrils.

##### RLC Protein Phosphorylation Determination

Samples were heated at 95 °C for 2 min to aid protein denaturation. Gels were subsequently loaded with 10-μl individual samples. Phos-tag^TM^ incorporating 15% SDS-PAGE and PVDF membrane blotting with chemiluminescent antibody detection were used to ascertain quantitative RLC phosphorylation levels. Gels were run for 120 min and blotted for 60 min. Membranes were probed with mouse anti-RLC antibodies (F109.3E1, Alexis Biochemicals, Nottingham, UK). Goat anti-mouse horseradish peroxidase (HRP)-linked IgG was used with a Bio-Rad electrochemiluminescence kit for band visualization in a Bio-Rad GelDoc imager. ImageJ was used to threshold and measure individual band densities.

##### Cloning and Expression of Recombinant RLC (rRLC)

rRLC was expressed from pET6a vector fused to an N-terminal histidine tag and tobacco etch virus protease site. After expression, cells harvested by centrifugation (10 min, 5000 × *g*) were lysed by BugBuster MasterMix (71456-4, Novagen) containing EDTA-free protease inhibitor mixture (11873580001, Roche Applied Science), and the inclusion bodies were purified according to the manufacturer's instructions. The purified inclusion bodies were resolubilized in IMAC binding buffer (25 mm HEPES, 500 mm NaCl, 1 mm MgCl_2_, 1 mm DTT, 20 mm imidazole) containing 6 m urea for 4 h at 4 °C under vigorous stirring. After removing insoluble components by centrifugation at 15,000 × *g* for 30 min, the supernatant was applied to a 5-ml HisTrap FF column (GE Healthcare) with a flow rate of 1–2 ml/min. The column was washed with 20 column volumes of IMAC binding buffer containing 6 m urea, and the protein was eluted with a step gradient of imidazole (50–500 mm, 50 mm increments) in IMAC binding buffer containing 6 m urea. Fractions containing purified protein were pooled, and urea were removed by dialyzing three times against dialysis buffer (25 mm HEPES, 250 mm NaCl, 1 mm MgCl_2_, 1 mm DTT). To remove the N-terminal histidine tag, tobacco etch virus protease was added in a 1:100 stoichiometry, and the protein was digested overnight at 4 °C. The sodium chloride concentration was adjusted to 500 mm, and tobacco etch virus proteases and remaining impurities were removed by passing through 1-ml HisTrapFF (GE Healthcare) columns. The column flow-through containing the purified rRLC was collected, and the protein was concentrated to 2 mg/ml (UFC901024, AMICON spin concentrators). Protein was stored at −80 °C until further use.

##### RLC Phosphorylation

rRLC phosphorylation species from *Rattus norvegicus* were created by incubation of rRLC with cardiac myosin light chain kinase donated by Mathias Gautel (King's College London) and zipper-interacting protein kinase (28) (Invitrogen) at 37 °C for 120 min in a kinase buffer (25 mm HEPES, 200 mm NaCl, 1 mm MgCl_2_, 1 mm DTT, 5% glycerol, and 200 μm ATP). Fresh kinase (1 μg/ml of reaction mixture) was added to the reaction mixture every 30 min. Kinases and phosphatases were removed by molecular weight cut-off column filtration. Phosphorylated rRLC was mixed 50:50 with unphosphorylated rRLC to produce control-mimicking *in vivo* RLC phosphorylation. Dephosphorylated rRLC was produced by incubation with shrimp alkaline phosphatase (Fermentas) (20 units/100 μl of protein) *in vitro* for 60 min in kinase buffer without ATP.

##### RLC Labeling and Exchange

Following expression, the buffer solution (25 mm HEPES, pH 7.1) containing recombinant RLC was exchanged to a labeling buffer (0.1 m NaCl and 0.1 m NaH_2_PO_4_ at pH 7.2) using PD-10 columns (GE Healthcare). Protein was subsequently incubated on ice for 2 h with *N*-hydroxysuccinimide-rhodamine ester (10 mg/ml in DMSO; Thermo Scientific). Excess dye was removed using Zeba^TM^ desalting spin columns (Thermo Scientific). Absorbance at 555 nm for the protein dye conjugate was used to indicate the labeling ratio of dye to protein. The buffer was changed to 5 mm ATP, 5 mm EGTA, 5 mm EDTA, 10 mm imidazole, 150 mm potassium proprionate, 10 mm KH_2_PO_4_, 5 mm DTT, and 0.5 mm trifluoperazine at pH 6.5 (exchange solution) for protein exchange as previously described (29–31). Explanted, permeabilized, and T-clipped trabeculae were mounted between two hooks at a sarcomere length of 2.1 μm and cooled in relaxing solution to 0.5 °C. Trabeculae were moved to a stage at 20 °C to be incubated for 45 min in 0.5 mg/ml RLC exchange solution, which incorporated 0.5 mg/ml TnC. TnC was added to all exchange solutions to reintroduce TnC that is lost during exchange so as not to alter contractile characteristics of the preparations. Trabeculae were incubated in three sequential 15-min incubations with fresh 30-μl drops of RLC exchange solution incorporating TnC. The preparation was then washed three times (15 min each, at 0.5 °C) in relaxing solution containing 0.5 mg/ml TnC to remove unbound RLC.

##### Force-Velocity Protocol

**FIGURE 1.**
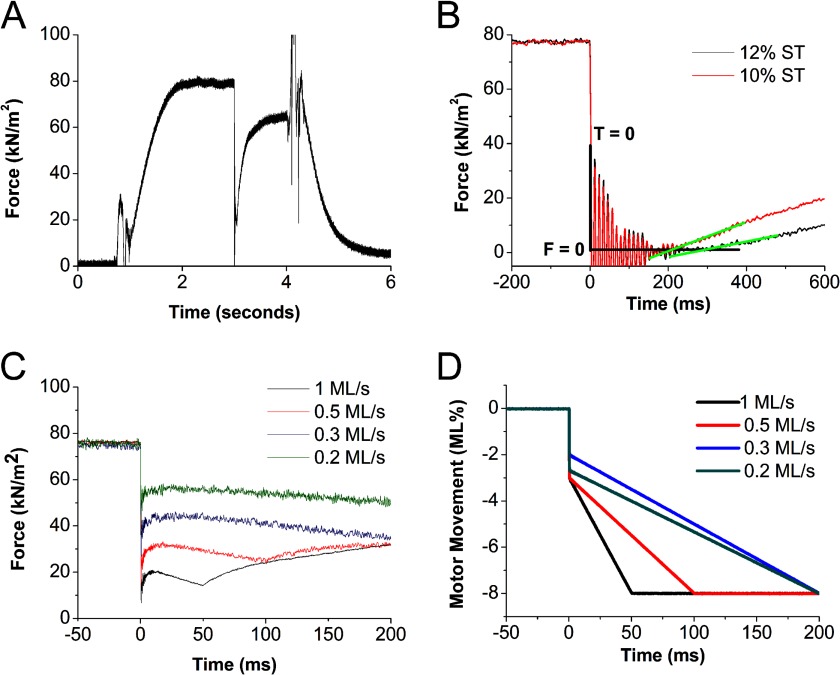
*A*, representative tension trace during a temperature jump activation between 0.5 and 20 °C of a permeabilized trabeculum. Force rises to a isometric plateau. 3 s after the temperature jump, the muscle is rapidly released and shortened at a fixed velocity for 50 ms, and then force recovers at the shortened sarcomere length. Muscle is then restretched to the original length and relaxed in a low calcium solution at 4 s. *B*, tension traces during two slack test (*ST*) measurements encompassing a 10% rapid release (*black trace*) and 12% rapid release (*red trace*). Time zero (*T* = 0) is reset to mark the time of the onset of the release. The time taken for tension to deviate from zero “slack force” (*F* = 0) in each slack length test is used to calculate peak unloaded shortening velocity (*V*_max_). Shown are tension traces during a range of fixed velocities (*C*) and the corresponding motor traces showing the release lengths and ramp velocities of each maneuver (*D*). *kN*, kilonewtons. *ML*, muscle lengths.

Trabeculae were mounted in relaxing solution between hooks attached at one end to a force transducer (AE801, HJK Sensoren + Systeme, Freidburg, Germany) and at the other to a motor (32). Shellac dissolved in ethanol was used to fix the trabecular tissue in place. Sarcomere length was set to 2.1 μm using diffraction of helium-neon laser light (633 nm) to visualize the first order diffraction pattern spacing. Trabeculae were taken through a sequence of solution changes using a two-stage quartz-surfaced platform system that was temperature-controlled (2 or 20 °C) and moved horizontally using a stepper motor (33). Each platform had two pedestals, which carried 30 μl of solution on their surface. The platform was moved so that the trabecular tissue was submerged in these bubbles of solutions in the following order: preactivating (2 °C), activating (2 °C), activating (20 °C), and relaxing (20 °C) representative tension trace shown in Fig. 1*A*. Trabecular dimensions were recorded by observation with transmitted light with a ×40 objective (0.75 numerical aperture, water; Zeiss). The stage movements and trabecular lengths were controlled using LabView software that also recorded digitized motor and force transducer outputs during experimentation. Upon activation and after peak isometric force was attained, “slack test” measurements on each preparation were carried out. Trabeculae were slackened by rapidly reducing length to between 8 and 12% of the initial muscle length. This was performed three times with each fiber. The time of force deviation from “slack force” was determined by fitting through the initial 100 ms of the tension recovery trace. The time taken for force to rise above the zero-force level was measured in each case by fitting the initial linear phase of tension development post-slack (Fig. 1*B*, *green lines*). *V*_max_ was calculated from the slope of the line joining the data points resulting from a plot of step length amplitude *versus* slack time (34). The order in which the slack lengths were applied was randomized. FV measurements incorporated release/ramp protocols (35) with ramp velocities between 0.1 and 1.5 muscle lengths/s. The ratio of muscle length in the ramp and in the release is dependent on the velocity of the ramp to achieve an isotonic plateau during the protocol. In our experiments, a rapid release of 8% of initial muscle length was applied (Fig. 1, *C* and *D*). The rapid release component effectively slackens the parallel elastic component of the preparation, giving rise to a nearly steady force level during ramp shortening.

##### Force-Velocity Relationships

Force measurements were normalized to trabecular cross-sectional area and reported as kilonewtons/m^2^. *V*_max_ is reported in muscle lengths/s. For each trabeculum, the relationship between force during shortening and shortening velocity was fit to a hyperbola previously described by Hill (36). The parameters from these fits are presented as means ± S.E.

##### Statistics

Two-way analysis of variance was used to make multiple comparisons between treatment groups in Table 1. For other analyses, one-way analysis of variance was used to test our experimental hypotheses. Appropriate post hoc pairwise multiple comparisons were made using a Bonferroni adjusted *t* test method with an overall significance of *p* < 0.05 in all instances.

**TABLE 1:** **Rat model of myocardial infarction shows compensated hypertrophy at 4 weeks with decompensation by 16 weeks** Heart weight/body weight ratios reveal a hypertrophic response at both time points compared with controls, although it is significantly greater at 4 weeks. Echocardiography reveals a reduced ejection fraction at both time points compared with controls, but it is further reduced by 16 weeks compared with 4 weeks post-MI. Numeric values for ejection fraction and heart weight/body weight ratios are stated as mean ± S.E. with *n* numbers accompanying each value. HW/BW, heart weight/body weight; AMC, age-matched control.

	Ejection fraction		HW/BW	
	AMC	MI*^a^*	AMC	MI
4 weeks	78.6 ± 3.89 (*n* = 9)	43.3 ± 3.89 (*n* = 9)	3.43 ± 0.17 (*n* = 9)	4.84 ± 0.17*^b^* (*n* = 9)
16 weeks*^c^*	74.5 ± 4.12 (*n* = *8*)	30.9 ± 3.89 (*n* = 9)	2.85 ± 0.18*^d^* (*n* = 8)	3.43 ± 0.17*^e^* (*n* = 9)

*^a^* Significant main MI treatment effect (*p* < 0.001).

*^b^* Significant MI effects, within 4-week pairing (*p* < 0.001).

*^c^* Significant main recovery time effect (*p* = 0.046).

*^d^* Significant recovery time effect within AMC pairing (*p* = 0.023).

*^e^* Significant MI effect within 16-week pairing (*p* = 0.024) and significant recovery time effect within MI pairing (*p* < 0.001).

## RESULTS

Phos-tag^TM^ gel electrophoresis was used to evaluate RLC phosphorylation levels during progression of a rat heart failure model caused by left anterior descending coronary artery ligation. A phosphorylation level of 0.53 ± 0.06 mol of P_i_/mol of RLC (*n* = 12) was observed in tissue from the inner left ventricular wall of the rat, in comparison with the phosphorylation level in non-failing human myocardium (0.41 ± 0.05 mol of P_i_/mol of RLC (*n* = 5)). These values are very similar to that of the pig left ventricular wall at 0.42 ± 0.02 mol of P_i_/mol of RLC (*n* = 2). Left ventricular RLC phosphorylation during the hypertrophic remodeling phase post-MI was 0.72 ± 0.03 mol of P_i_/mol of RLC (*n* = 5) *versus* healthy rat at 0.53 ± 0.06 mol P_i_/mol of RLC (*n* = 12), and this difference was assessed as the model progressed to chronically dilated and failing phenotype (0.76 ± 0.07 mol of P_i_/mol of RLC (*n* = 4), *p* < 0.05), showing a significant difference in late stage disease.

**FIGURE 2.**
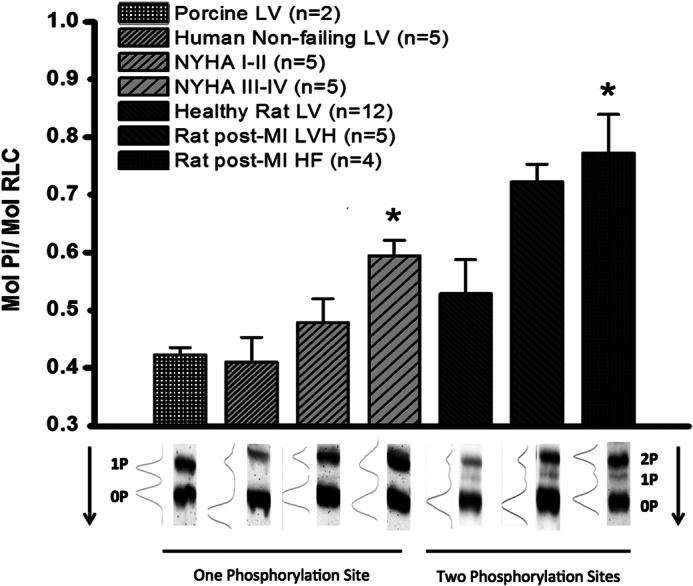
*Top*, SDS-PAGE Phos-tag^TM^ analysis of cRLC phosphorylation level in mol of phosphate/mol of cRLC. cRLC phosphorylation was measured in left ventricular tissue from porcine, human, and rat samples. Samples from human left ventricular tissue taken from patients were categorized as moderate (NYHA I or II (*n* = 5)) or severe heart failure (NYHA III or IV (*n* = 5)) or normal donor (*Human Non-failing LV*, *n* = 5). Normal rat left ventricular tissue (*Healthy Rat LV*, *n* = 12) was compared with rat LV 4 weeks post-chronic MI (*Rat post-MI LVH*, *n* = 5) and 20 weeks post-chronic MI (*Rat post-MI HF*, *n* = 4). All data are displayed as mean ± S.E. (*error bars*). *, significance of *p* < 0.05 compared with control. *Bottom*, representative Western blots showing the ratio of phosphorylation and the amount of phosphorylation sites in each organism. *Arrow*, direction of blot. *2P*, *1P*, and *0P*, the number of RLC sites phosphorylated for each band.

In addition, the RLC phosphorylation levels during progression of clinical heart failure in human patients as defined by symptomatic classification of severity (the NYHA classification) were evaluated. A similar trend of increased RLC phosphorylation during disease was found when we studied human heart failure tissue during progressive clinically designated disease severity. Those classified NYHA I and II show a similar trend, increasing during disease progression from a control phosphorylation state of 0.47 ± 0.05 mol of P_i_/mol of RLC (*n* = 4) to NYHA III and IV patient tissues, which show a significant increase, 0.6 ± 0.03 mol of P_i_/mol of RLC (*n* = 4) (*p* < 0.05) in comparison (Fig. 2).

In order to study the effect of RLC phosphorylation under more controlled conditions, cardiac RLC was expressed in bacteria. The phosphorylation levels of the RLC were increased by incubation with cardiac myosin light chain kinase and zipper-interacting protein kinase. The Phos-tag^TM^ gels indicated that a total of ∼1 mol of P_i_/mol of RLC is incorporated into the RLC by this treatment. Another fraction of the expressed RLC was incubated with the nonspecific phosphatase shrimp alkaline phosphatase to ensure that all RLC species were dephosphorylated.

**FIGURE 3.**
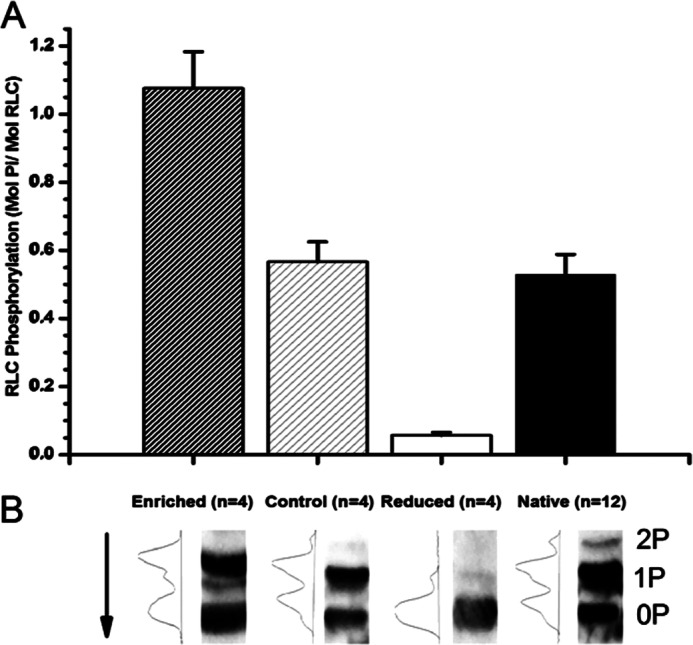
*A*, *bar plot* indicating the phosphorylation level of differently phosphorylated rat rRLC species generated by incubation with kinases (*Enriched*), shrimp alkaline phosphatase (*Reduced*), or a mixture of the two mimicking native RLC phosphorylation levels (*Control*). Native trabecular phosphorylation level is displayed for comparison (*Native*). All data are plotted as mean ± S.E. (*error bars*), and *n* values are given for each preparation. *B*, representative Western blots of differently phosphorylated rRLC species. *Arrow*, direction of blot. *2P*, *1P*, and *0P*, number of RLC sites phosphorylated.

These RLCs could be exchanged into skinned rat trabeculum fibers to replace the exogenous RLCs. Exchange treatments to mimic the “endogenous” levels used a mixture of the *in vitro* phosphorylated and dephosphorylated light chains to achieve a total phosphorylation level of 0.5 mol of P_i_/mol. A “reduced” phosphorylation level was achieved by exchange of the dephosphorylated RLC alone, and an “increased” RLC phosphorylation level was achieved by using the phosphorylated RLC fraction for the exchange. These differently phosphorylated rRLC species were compared with endogenous trabecular RLC “native” phosphorylation (Fig. 3).

**FIGURE 4.**
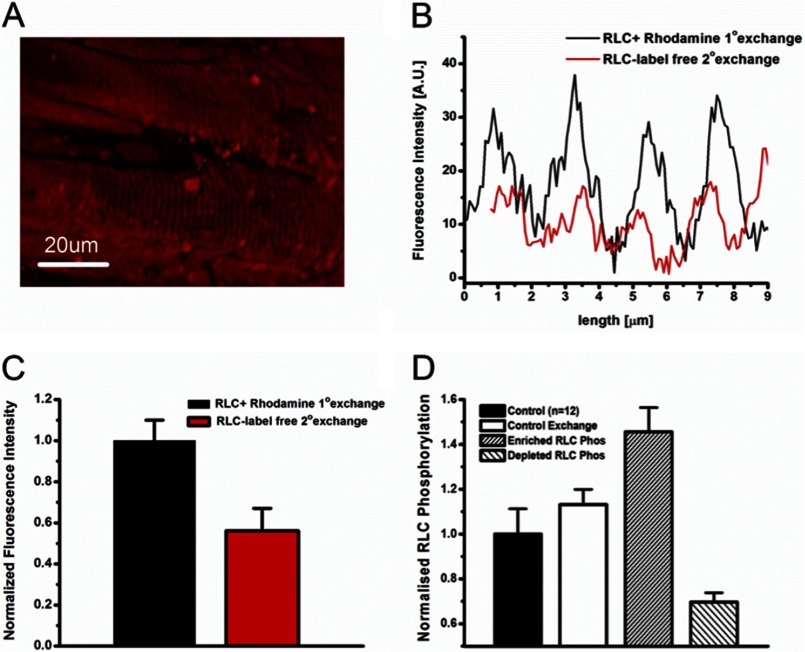
*A*, confocal image of a permeabilized cardiac trabeculum after exchange with a recombinant fluorescently labeled rRLC. *B*, fluorescent intensity across sarcomeres measured before and after a secondary exchange with an unlabeled recombinant RLC. *C*, exchange efficiency calculated from integrating fluorescent intensity before and after secondary exchange. Data presented are an average ± S.E. of *n* = 3 separate exchange procedures on permeabilized trabeculae. *D*, calculated RLC phosphorylation changes after trabeculae have undergone exchange with differently phosphorylated rRLC species. *Control*, the native left ventricular phosphorylation level of RLC (*n* = 12). *Control Exchange*, the calculated phosphorylation level of trabeculae that have undergone exchange with rRLC that is similarly phosphorylated to native RLC. *Enriched RLC Phos*, the RLC phosphorylation after exchange with enriched rRLC species. *Depleted RLC Phos*, the effect on trabecular phosphorylation after exchange with dephosphorylated rRLC species. *Error bars* are calculated from S.E. values of exchange efficiencies.

Fluorescently labeled RLC was exchanged into permeabilized trabecular muscle preparations to visualize the spatial incorporation of these recombinant RLC species into the sarcomeric network. Fig. 4*A* shows a three-dimensional *z* stack reconstruction of the surface of a cardiac trabeculum that has undergone exchange with rhodamine-labeled RLC. We show specific localization of recombinant labeled RLC to the myosin-containing A-band region, and only background levels of fluorescence in the myosin-lacking I-band regions (Fig. 4*B*). The A-band region was measured as 1.69 ± 0.05 μm at a sarcomere length of 2.25 ± 0.02, consistent with literature values for A-band length (*n* = 3) (37, 38). Exchange efficiency was analyzed in skinned trabecular samples by performing a secondary exchange with unlabeled recombinant RLC as a “washout” protocol for fluorescently labeled RLC species. Fig. 4*B* presents an example of fluorescence intensity profile across many sarcomeres showing a significant fluorescence decrease after the secondary exchange with unlabeled RLC. 50% exchange efficiency was evaluated (Fig. 4*C*) by comparing pre- and post-washout fluorescence profiles. This allowed the estimation of RLC phosphorylation state postexchange for trabeculae treated with enriched or dephosphorylated recombinant RLC species (Fig. 4*D*).

**FIGURE 5.**
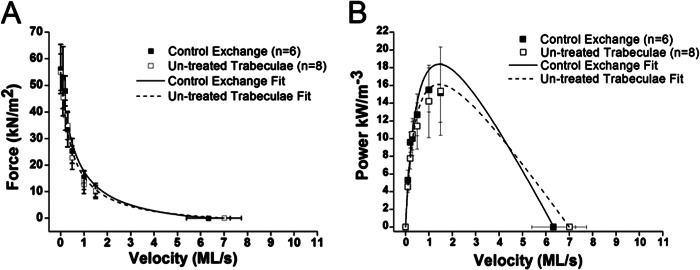
*A*, FV relationships of untreated trabeculae (*n* = 8) compared with trabeculae that have undergone control exchange (*n* = 6) with rRLC species of similar to native levels of RLC phosphorylation. Force was measured during the plateau phase for the individual fixed shortening velocity. Relations are fit with Hill's hyperbolic equation. All points are means of force produced during shortening ± S.E. (*error bars*). *B*, PV relations calculated from FV relations of untreated trabeculae (*n* = 8) compared with control exchange (*n* = 6). All data are presented as power ± S.E. This fitting procedure fits the relationship between force during shortening and shortening velocity in muscle lengths (*ML*)/s. Microsoft Excel solver was used to minimize the sum of the squared error between observed and expected values of force. The V_max_ measurements obtained by the slack test method constrain the fitting procedure.

Postexchange *V*_max_ values (derived from slack tests) and force measurements at different release velocities were combined to determine the FV relations (see Fig. 1) for each group of trabeculae (dephosphorylated, enriched phosphorylation, control). Control-exchanged trabeculae were compared with untreated trabeculae to assess the effect of RLC exchange (Fig. 5, *A* and *B*). These two groups did not show significant differences in most parameters (Table 2). However, the curvature of Hill's hyperbolic fit (*a*/*P*_o_) of the FV relations was affected by control exchange. Control exchange showed a 2-fold increase in the *a*/*P*_o_ parameter (*p* < 0.05).

**TABLE 2:** **Numeric values of parameters from FV and PV fits** All values are displayed as mean ± S.E.

	Enriched (*n* = 8)	Control (*n* = 6)	Native (*n* = 8)	Dephosphorylated (*n* = 5)
Peak isometric force (kilonewtons/m^2^)	81.9 ± 10*^a^*	79.9 ± 11	86.2 ± 9*^a^*	22.3 ± 7
*V*_max_ (muscle lengths/s)	8.85 ± 0.7*^a^*	6.33 ± 0.9	7.01 ± 0.7	5.34 ± 0.4
*a*/*P*_o_	0.16 ± 0.06	0.1 ± 0.02	0.04 ± 0.01	0.07 ± 0.02
Power (kW/m^−3^)	38.9 ± 8*^a,b^*	17 ± 3	14.7 ± 4	5.13 ± 3
Velocity at peak power (muscle lengths/s)	1.97 ± 0.2*^a,b^*	1.36 ± 0.1	1.03 ± 0.2	0.9 ± 0.2

*^a^* Different from dephosphorylated (0.02 < *p* < 0.001).

*^b^* Different from native (*p* < 0.05).

**FIGURE 6.**
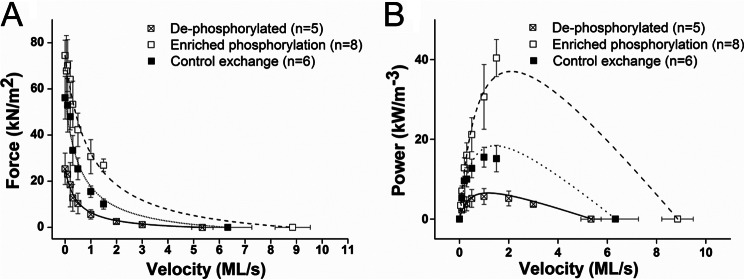
*A*, FV relationships of trabeculae that have undergone exchange with rRLC species that are hyperphosphorylated (*n* = 8), have control phosphorylation similar to native RLC phosphorylation (*n* = 6), and are dephosphorylated (*n* = 5). *B*, calculated PV relationships of trabeculae that have undergone exchange with the three species of rRLC phosphorylation. All data are plotted as mean ± S.E. (*error bars*). *ML/s*, muscle lengths/s.

**FIGURE 7.**
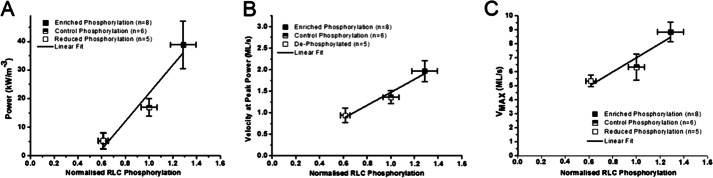
**Parameters from hyperbolic fits of FV and PV relationships plotted as mean ± S.E. (*error bars*) by RLC phosphorylation magnitude normalized to control phosphorylation.** All parameters that had significant differences between phosphorylation statuses were plotted: power (*A*), velocity at peak power (*B*), and peak unloaded shortening velocity (*V*_max_) (*C*). All data sets were described with a linear fit.

RLC phosphorylation state most clearly altered the force, power, and *V*_max_ of trabeculae (Table 2 and Fig. 6). Mean force and power in the enriched RLC phosphorylation trabeculae were >3.7- and 7.5-fold higher, respectively, than mean force and power in the reduced phosphorylation series (*p* < 0.005). Average *V*_max_ was 66% higher in the phosphorylation-enhanced trabeculae compared with the reduced phosphorylation state (*p* = 0.02). The multiple-comparison statistical models did not always yield significant differences between the control group, which consisted of a 50:50 dephosphorylated/phosphorylated exchange mixture, and the reduced and enhanced phosphorylation groups (Table 2). For instance, power output in the phosphorylation-enriched group was marginally different from the peak power observed in the control and native phosphorylation groups (0.08 < *p* < 0.04). The velocity at which peak power occurred (*V*_PP_) showed a similar pattern; *V*_PP_ in the enhanced phosphorylation group was >2-fold faster than in the reduced phosphorylation state (*p* = 0.012) and showed a marginal difference from the “native” control (*p* = 0.04). The mean force, power, and *V*_max_ for the 50:50 control exchange series were consistently intermediate to the reduced and enhanced phosphorylation groups (Figs. 6 and 7). RLC phosphorylation state seems to be a key regulator of a continuum of physiologically relevant aspects of cardiac performance.

## DISCUSSION

In this study, we have evaluated the effect that altering cRLC phosphorylation has on cardiac muscle mechanics. We observe clear modulation of cardiac muscle function by RLC phosphorylation. The alteration of many mechanical properties in our experiments provides evidence that RLC phosphorylation state is an important modulator of the behavior of cardiac muscle in health and disease.

It is known that RLC phosphorylation does not change significantly between systolic and diastolic states (39), but changes to phosphorylation may accompany cardiac fitness, pathology, and/or age. We have shown a dramatic increase in RLC phosphorylation in both a model of chronic myocardial infarction in rats and during the progression of NYHA-categorized HF in human myectomy samples (Fig. 2). We demonstrate a correlation between phosphorylation change of the RLC and cardiac performance that is conserved between rat and human cardiac tissue during HF progression. This observation is complementary to the evidence that RLC phosphorylation can drive cardiac hypertrophy (21). Reductions in RLC phosphorylation due to RLC mutations, expression of non-phosphorylatable RLC, or during human heart failure are also observed (12, 13, 19, 20, 22, 25, 26, 40–42). Interestingly, decreases in RLC phosphorylation have been observed when the catalytic subunit of the type 1 protein phosphatase δ isoform (PP1cδ) first identified by Nishio *et al.* (43) and MYPT2 (regulatory subunit) have been transferred into neonatal cardiomyocytes, which coincided with blocked sarcomere organization (43, 44). PP1cδ activity stimulated by overexpression of MYPT2 showed decreased basal RLC phosphorylation, reduced contractility, and a phenotype of left ventricular hypertrophy (45).

However, it is important to note that human and rat RLCs are different. Human RLC comes as two distinct charge variants with identical molecular weight, denoted as LC-2 and LC-2* (8). The LC-2 isoform is more highly expressed (70% LC-2/30% LC-2*) and more highly phosphorylated (8, 41). This ratio of expression does not often seem to alter during disease (8). Therefore, human ventricular tissue exists as three different myosin isoenzymes (LC-2/LC-2, LC-2/LC-2*, and LC-2*/LC-2*). This does not hold true for rats, where one ventricular isoform of RLC is evident and the three isoenzymes are formed as mixtures of α and β myosin heavy chains (46). These human ventricular RLCs possess single serine phosphorylation sites, whereas the single rat ventricular isoform of RLC has two phosphorylatable sites (11, 26, 47). The possible function of this second site is so far unknown. It would certainly be of interest to know the effect that the second phosphorylation site has on myosin structure and function, specifically whether this second site is complementary to the function of the first. The relationship between RLC phosphorylation change and cardiac performance that we observe in myectomy samples and in our rat model is conserved in our human samples, suggesting that phosphorylation has a similar role, whether RLC is mono- or diphosphorylated. Interestingly, diphosphorylated RLC species are present in animals in which α-cardiac myosin heavy chain predominates and where the heart rate is fast. Species with predominance toward β-cardiac myosin heavy chain and lower heart rates possess a single phosphorylatable site and two ventricular RLC isoforms.

The exact role of RLC as a modulator of cardiac muscle and the mechanisms by which it is regulated have been debated (39, 48). Indirect evidence provided by electron microscopy and optical diffraction studies have supported the hypothesis that RLC phosphorylation alters the disposition of myosin heads by charge repulsion away from the thick filament (14–16, 49). The use of isolated myosins highlights a second hypothesis that involves the elasticity of the lever arm region, whereby phosphorylation increases stiffness, increasing the myosin duty cycle (17, 18). As of yet, the direct effect of cRLC phosphorylation on cardiac muscle performance during shortening at intermediate velocities where the muscle is performing mechanical work has not been studied. Only *V*_max_ and peak isometric force have been evaluated (50–53), and the results are similar to those observed in the present study.

We show that we can alter RLC phosphorylation with the use of kinases (cardiac myosin light chain kinase and zipper-interacting protein kinase) *in vitro* (Fig. 3) and exchange these phosphorylated species into permeabilized cardiac preparations to alter RLC phosphorylation (Fig. 4). Importantly, the alterations to phosphorylation we have made are within the range of the phosphorylation changes observed during disease. We observed up to 0.8 mol of P_i_/mol of RLC in failing rat myocardium and increased RLC phosphorylation in our experimental treatments to ∼0.7 mol of P_i_/mol of RLC. Previously reported RLC phosphorylation in disease phenotypes can be completely abolished and significantly reduced (12, 19, 20, 54, 55). We have reduced phosphorylation significantly to ∼0.2 mol of P_i_/mol of RLC.

We show that our exchange protocol used to replace native cRLC with rRLC did not affect the FV and PV relations of the trabecular preparations (Table 2 and Fig. 5), demonstrating that the RLC exchange protocol had little effect on the mechanical function of the permeabilized trabeculae. Hence, our results are fully accounted for by the altered RLC phosphorylation.

The range of differently phosphorylated RLC preparations showed large changes to force and power during shortening when compared with control (Fig. 6). Interestingly, a doselike response of RLC phosphorylation affected many mechanical parameters, such as the velocity at which peak power was attained (*V*_PP_), *V*_max_, and power (Fig. 7). Clearly, RLC phosphorylation level affects the ability of cardiac muscle to produce power during shortening that is not only a calcium sensitivity effect (19, 22–24) because these effects are demonstrated in saturating 32 μm [Ca^2+^].

To our knowledge, the effect of RLC phosphorylation during shortening at intermediate, work-producing velocities has not been studied, and hence power production by the myocardium has not been assessed. The power produced during shortening is an important factor to study because it can be related to cardiac muscle function at different heart rates. Our observations together with these early findings define two distinct roles for RLC phosphorylation: 1) the inability of the RLC to be phosphorylated is a driver for cardiac pathology; 2) increases in RLC phosphorylation may signify a compensatory adaptation to pathology in certain instances.

Our results complement others that suggest that cardiac disease progression may be driven by an inability to phosphorylate RLC appropriately. Here we extend the role of RLC phosphorylation in pathophysiology to acquired heart failure post-MI, with confirmation in myocardial samples from patients with advanced heart failure and an animal model of chronic MI in rats. Thus, increased RLC phosphorylation may act as a mechanism to compensate for the loss of myofilament mechanical power generation during progression of the failing myocardium.

## Footnotes

2 The abbreviations used are:

HF: heart failure
FV: force-velocity
MI: myocardial infarction
NYHA: New York Heart Association
PV: power-velocity
RLC: regulatory light chain
rRLC: recombinant regulatory light chain
TnC: troponin C.
